# Case Report: Infection with *Streptococcus constellatus* manifesting as gelatinous pleural effusion in an HIV-positive patient

**DOI:** 10.3389/fimmu.2025.1545165

**Published:** 2025-03-18

**Authors:** Zhongxia Yang, Wanyuan Xiong, Zibing Qian, Xiaorong Mao

**Affiliations:** ^1^ Department of Infectious Diseases, The First Hospital of Lanzhou University, Lanzhou, Gansu, China; ^2^ The First School of Clinical Medicine, Lanzhou University, Lanzhou, Gansu, China

**Keywords:** *Streptococcus constellatus*, gelatinous, pleural effusion, HIV, infection

## Abstract

*Streptococcus constellatus*, recognized as a commensal bacterium, has the potential to induce severe infections in patients with immunodeficiency. Here, we reported a rare case of *Streptococcus constellatus* infection that manifested as gelatinous pleural effusion in a patient with HIV. Although the effusion was gelatinous and partially encapsulated, it was completely resolved with timely administration of antibiotics, thereby eliminating the necessity for thoracic drainage, urokinase injection, or surgical intervention.

## Introduction

*Streptococcus constellatus* is found in various areas of the human body, such as the skin, nasopharynx, stomach, and intestines. When the immune system is compromised by various factors, *Streptococcus constellatus* can become pathogen. Patients infected with human immunodeficiency virus (HIV) are especially vulnerable to opportunistic infections due to chronic inflammation and T-cell dysfunction. Several cases have reported that HIV-positive patients present with brain abscesses (1), muscle abscesses (1), liver abscesses (2), and pleural effusions (3) attributed to *Streptococcus constellatus*.

Pleural fluid resulting from *Streptococcus constellatus* is predominantly purulent in nature and can be easily encapsulated (3). To the best of our knowledge, only one case reported that *Streptococcus constellatus* leaded to pleural fluid in patient with HIV, while the characteristics of fluid was milky white (3).

It is well known that jelly-like effusions primarily occur in the patients with malignant tumors, and these cases are challenging to treat and often resulting in a poor prognosis (4). There have been no documented cases of jelly-like effusions linked to *Streptococcus constellatus*. The case highlights the importance of identifying atypical clinical presentations, such as gelatinous pleural effusion, which may indicated an underlying bacterial infection.

## Case description

A 58-year-old male patient was diagnosed with HIV approximately one year ago. He has been receiving antiretroviral therapy consisting of lamivudine, tenofovir disoproxil fumarate, and efavirenz. His medical history includes type 2 diabetes and a tooth extraction.

**Figure 1:**
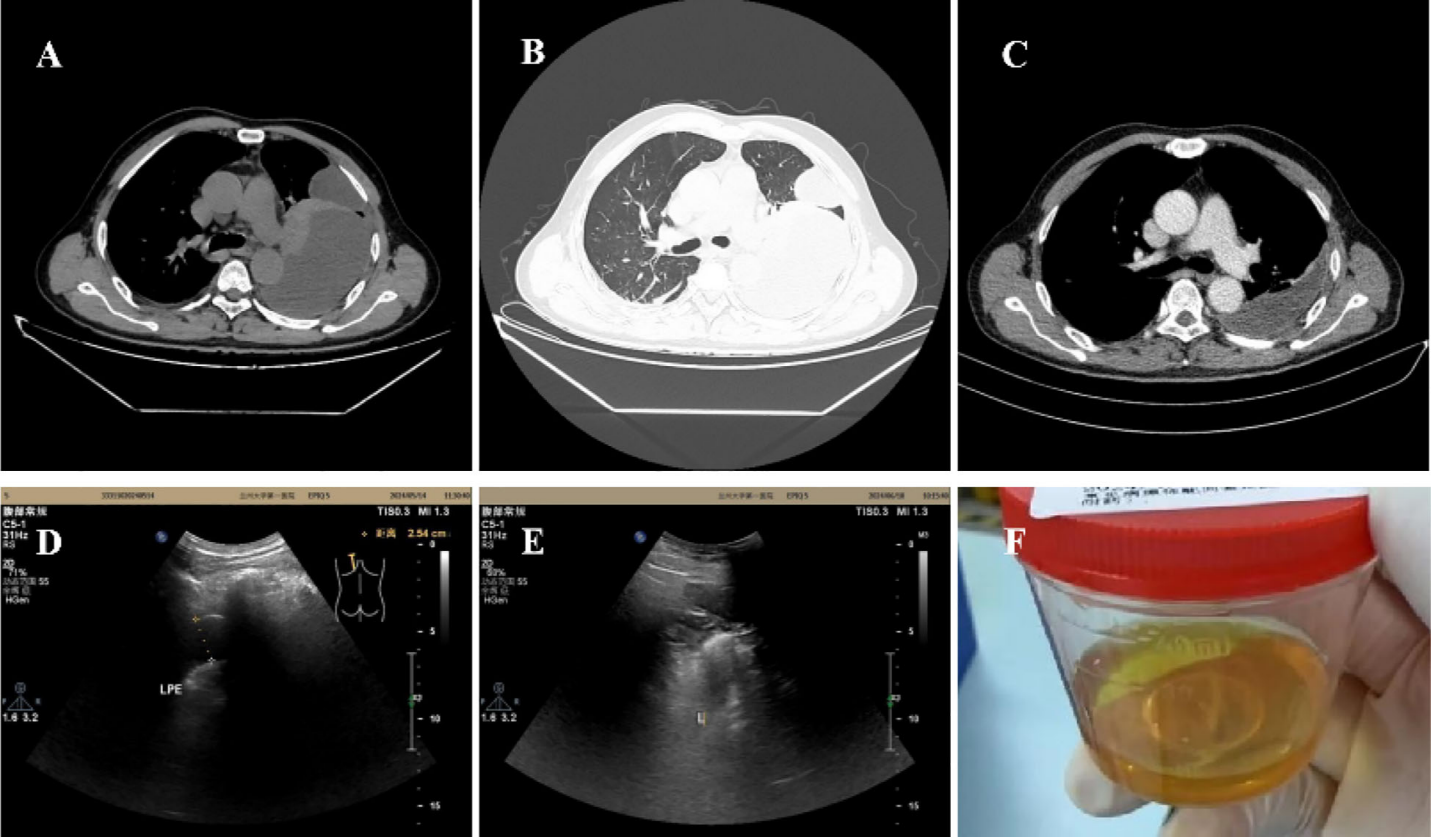
**(A, B)** On admission, CT showed a left-sided pleural effusion, partially encapsulated. **(C)** CT showed pleural effusion was signaficatlly decreased 15 days after treatment. **(D)** 30 days after discharge, ultrasound showed the left thoracic cavity (posterior axillary line, scapula level) had 10 mm and 25 mm pleural effusion, respectively. **(E)** Pleural effusion was completely absorbed 66days after discharge. **(F)** Jelly-like pleural fluid was withdrawn on admission.

The patient was admitted to our hospital with hyperpyrexia, shortness of breath, chest pain, and chest tightness, symptoms that had persisted for seven days. The highest recorded body temperature was 39°C, without chills, cough, or expectoration. Upon admission, his vital signs were as follows: temperature 38.7°C, heart rate 107 beats per minute, respiratory rate 20 breaths per minute, blood pressure 135/98 mmHg, and oxygen saturation 87% on room air. Percussion of the left lung revealed dullness, and auscultation indicated absent breath sounds. The laboratory results were as follows: white blood cell (WBC) count was 12.81 x 10^9/L, neutrophil ratio was 84.9%, and lymphocyte ratio was 29.6%. C-reactive protein was 122.7 mg/L, and procalcitonin was 0.060 ng/mL. The erythrocyte sedimentation rate was 57 mm/h. Tumor markers, γ-interferon release test, and blood culture were negative. Ferritin level was 609.00 ng/mL. The CD4+ T cell count was 437 cells/µL, with a CD4/CD8 ratio of 0.56. Chest CT revealed pneumonia, left-sided pleural effusion, and partial encapsulation (**Figures 1A, B**). Approximately 50 mL of yellow, jelly-like pleural effusion was extracted from the left pleural cavity (**Figure 1F**). Effusion was sticky and difficult to remove. The results of the effusion analysis showed an erythrocyte count of 31,900 x 10^6/L, a WBC count of 46 x 10^6/L, a negative mucin test, a glucose level of 7.77 mmol/L, an LDH level of 1,266 U/L, and an adenosine deaminase level of 23.50 U/L. The pleural effusion culture, acid-fast staining, and tuberculosis bacteria GeneXpert tests were all negative. The metagenomic next-generation sequencing (mNGS) of the pleural effusion identified *Streptococcus constellatus*.

The diagnoses were: 1. Pleural effusion, 2. Pneumonia, 3. AIDS, 4. Type 2 diabetes. Anti-infective treatment was piperacillin sodium and tazobactam sodium 4.5 g, administered intravenously every 12 hours. The symptoms were significantly relieved and CT showed that the pleural effusion had decreased significantly after therapy (**Figure 1C**). The patient was discharged from the hospital and continued medication treatment after 16 days. The antibiotics were adjusted to nemonoxacin malate tablets 0.5 g, taken once daily by mouth. The pleural fluid was gradually absorbed (**Figure 1D**). After 66 days post-discharge, a follow-up ultrasound showed that the pleural effusion had disappeared (**Figure 1E**). The changes in vital signs and laboratory indicators during the patient’s course of illness are presented in **Table 1**.

**Table 1:** Changes in the patient's vital signs and laboratory indicators.

	2024.3.20	2024.3.27^*^	2024.4.1	2024.4.7	2024.4.23	2024.6.18
Temperature (°C)	39.0	38.7	37.4	36.7	36.5	36.9
Heart Rate (beats per minute)	117	107	108	100	88	–
Respiratory Rate (beats per minute)	22	20	19	20	18	–
Blood Pressure (mmHg)	130/80	135/98	111/73	105/78	110/74	–
Oxygen Saturation (%)	57.6	87	89	93	98	99
WBC (x 10^9/L)	15.33	12.81	7.47	7.81	6.18	7.47
N (%)	87.6	84.9	65.8	63.60	56.7	65.8
PLT (x 10^9/L)	176	345	168	389	226	168
HGB (g/L)	155	142	164	163	155	164
ALT (U/L)	12	32	76	63	34	29
AST (U/L)	16	42	61.1	41.6	23	21
TBIL (μmol/L)	11.6	10.2	7.9	6.3	7.9	7.8
ALB (g/L)	41.7	37.8	33.7	29.5	45.7	46.6
CRP (mg/L)	87.5	122.7	156.84	–	5.15	1.52
Cr(umol/L)	54.6	51.2	78	65	64	61
BUN (mmol/L)	5.16	3.24	5.0	4.36	3.3	3.9
Blood sugar (mmol/L)	11.94	10.75	9.84	6.77	6.23	5.96
PT (s)	13.3	13.9	14	15.85	9.6	–
ESR (mm/h)	87.5	57	58	44	–	–

*indicates the time of the patient's admission. WBC, white blood cell; N, neutrophil ratio; PLT, platelet; HGB, hemoglobin; ALT, Alanine aminotransferase; AST, Aspartate aminotransferase; TBIL, Total bilirubin; ALB, Albumin; CRP, C reactive protein; Cr, Creatinine; BUN, Urea Nitrogen; PT, Prothrombin time; ESR, Erythrocyte sedimentation rate. “-” iindicates that the test was not conducted.

## Discussion and conclusions

*Streptococcus constellatus* is considered as a pathogenic bacterium capable of causing abscesses, deep-seated inflammation, and vascular lesions, including brain abscesses (5), liver abscesses (6), pneumonia (7), lung abscesses (8), empyema (3), descending necrotizing mediastinitis (9), pelvic inflammatory disease (10), suppurative thrombophlebitis (11), venous sinus thrombosis (12), and secondary bone destruction (13). Infections caused by *Streptococcus constellatus* are diverse in nature and are strongly associated with compromised host immunity. The current case presented as pleural effusion attributed to *Streptococcus constellatus*, with the underlying medical condition being HIV.

It is essential to identify the nature of effusion to improve therapeutic outcomes. Typically, the appearance of effusion caused by *Streptococcus constellatus* is purulent, turbid, or milky white (3)and is prone to forming loculated effusions. However, the appearance of the pleural effusion in our case was jelly-like, which is not consistent with other patients. The jelly-like effusion primarily originates from malignant tumors, such as mesothelioma (4) and melanoma (14), and is occasionally found in infections caused by tuberculous bacilli (15, 16). The gelatinization of pleural fluid indicates a higher viscosity, primarily linked to the content of mucoproteins and hyaluronic acid in the effusion (15–19). Malignant cells may produce large amounts of mucin. Additionally, hyaluronic acid has been found in pleural effusions caused by malignant tumors and inflammatory pleural diseases (15–17, 19). Patients with pleural metastatic melanoma may develop pleural effusion (20). Here, we reported the first case of jelly-like effusion caused by a *Streptococcus constellatus* infection.

The pathogenicity of *Streptococcus constellatus* is also associated with the disruption of the barrier at the colonization site. *Streptococcus constellatus* may originate from the oral cavity, esophagus, stomach, hepatobiliary system, or urinary tract. The onset of this case may be linked to a history of tooth extraction.

The duration of antibiotic treatment is often prolonged due to the tendency of *Streptococcus constellatus* to cause deep infections. If pleural effusion is not treated promptly, it can worsen, leading to complications such as effusion encapsulation and even pleural adhesion. Thoracotomy or thoracoscopic surgery can significantly increase both the psychological and financial burden on patients. Early diagnosis and timely administration of sensitive antibiotics can simplify the treatment, reduce the duration of the illness and hospitalization, and enhance the cure rate. In our case, the pathogen was promptly identified using metagenomic next-generation sequencing (mNGS), and the patient received a prolonged course of the appropriate antibiotic. As a result, the patient’s pyothorax was successfully treated, avoiding more complex interventions such as thoracic drainage, urokinase injection, and surgery.

In summary, *Streptococcus constellatus* is a pathogenic bacterium that can cause pleural effusion in patients with HIV/AIDS, and this pleural effusion may present with gelatinous characteristics. Early diagnosis and a timely, complete course of antimicrobial therapy can effectively treat gelatinous pleural effusion and may help the need for surgery.

## Data Availability

The original contributions presented in the study are included in the article/supplementary material. Further inquiries can be directed to the corresponding author/s.
